# Functional characterization of a SNP (F51S) found in human alpha 1‐antitrypsin

**DOI:** 10.1002/mgg3.819

**Published:** 2019-06-28

**Authors:** Hong‐Nhung Trinh, Sei‐Heon Jang, ChangWoo Lee

**Affiliations:** ^1^ Department of Biomedical Science and Center for Bio‐Nanomaterials Daegu University Gyeongsan South Korea

**Keywords:** Alpha 1‐antitrypsin deficiency, F51S, polymerization, single nucleotide polymorphism, thermal stability

## Abstract

**Background:**

Alpha 1‐antitrypsin (A1AT) deficiency is related to lung and liver diseases, including pulmonary emphysema and liver cirrhosis in humans. Genetic variations including single nucleotide polymorphisms (SNPs) of *SERPINA1* are responsible for A1AT deficiency, but the characteristics of the SNPs are not well‐understood. Here, we investigated the features of a rare SNP (F51S) of A1AT, which introduces an additional N‐glycosylation site in the N‐terminal region of A1AT.

**Methods:**

We evaluated the F51S variant compared with the wild‐type (WT) A1AT with regard to expression in CHO‐K1 cells, trypsin inhibitory activity, polymerization, and thermal stability.

**Results:**

The recombinant F51S protein expressed in CHO‐K1 cells was mostly retained inside cells. The F51S variant had trypsin inhibitory activity, but reduced thermal stability compared with the WT A1AT. The native acrylamide gel data showed that F51S tended to prevent polymerization of A1AT.

**Conclusion:**

The results of this study indicate that Phe51 and the surrounding hydrophobic residue cluster plays an important role in the conformation and secretion of A1AT and suggest the harmful effects of a rare F51S SNP in human health.

## 1 INTRODUCTION

Human alpha 1‐antitrypsin (A1AT), which is encoded by *SERPINA1* (OMIM entry: *107,400) located on the long arm of the chromosome 14 (14q32.13), is the most abundant serine protease inhibitor in the plasma (Seixas et al., 2001) and mainly inhibits neutrophil elastase in the lower respiratory tract (Gadek, Klein, Holland, & Crystal, 1981). The normal concentration of A1AT in human plasma ranges from 100 to 350 mg/dl (Allen, Ward, & Perks, 1986; Ferrarotti et al., 2012). Low concentrations of A1AT (<100 mg/ml) lead to A1AT deficiency in humans with liver and lung diseases, including liver cirrhosis, pulmonary emphysema, bronchiectasis, asthma, and cryptogenic fibrosing alveolitis (Fregonese & Stolk, 2008; Mahadeva, Stewart, Bilton, & Lomas, 1998).

Genetic variations, especially single nucleotide polymorphisms (SNPs) of *SERPINA1*, are mainly responsible for A1AT deficiency. Among 25 genetic variants of A1AT that have been investigated (Salahuddin, 2010), 16 SNPs are predisposed to A1AT deficiency (Ferrarotti et al., 2012; Fujimoto et al., 2010; Obeidat et al., 2011). The G67E variant resulting from a single point mutation of GGG to GAG (named Mmineral springs) led to reduced A1AT secretion levels in blood monocytes (Curiel, Vogelmeier, Hubbard, Stier, & Crystal, 1990). Another A1AT variant, E342K, formed insoluble polymers in the hepatocyte endoplasmic reticulum and, as a result, the amount of secreted E342K was reduced (Cox, Woo, & Mansfield, 1985). The SNPs are also responsible for chronic obstructive pulmonary disease (COPD) as a result of A1AT deficiency. Six haplotypes with a common backbone of five SNPs of *SERPINA1* were found to increase the risk of COPD up to 50‐fold (Chappell et al., 2006). Additionally, SNPs of genes other than *SERPINA1* were shown to be associated with the plasma level of A1AT (Setoh et al., 2015). Moreover, there are still over 500 SNP clusters in the coding region of *SERPINA1* that have been identified by human genetic variation projects; however, clinical details regarding the SNPs are unknown.

There are two main methods to investigate the effects of A1AT variants, case reports or genome‐wide association methods. In the case report method, the A1AT genes of patients with abnormal plasma A1AT levels are sequenced to identify genetic abnormalities (Allen et al., 1986; Graham et al., 1989; Hofker et al., 1989). The genome‐wide association study is an approach in which the genetic markers from a community‐dwelling population or a number of patients are scanned and their A1AT statuses are combined to identify the association between genetic variants and A1AT relevant diseases (Obeidat et al., 2011; Setoh et al., 2015). Although widely used in the detection of A1AT variants, none of these methods allows direct access of a particular known SNP in A1AT structure and function. Recently, a number of predictors have been developed to quickly scan and predict the harmful effects of novel SNPs (Niroula & Vihinen, 2016). However, the accuracy of each prediction algorism must be verified by in vivo or in vitro characterization (Giacopuzzi et al., 2018).

There are notable hydrophobic residues located on β‐strand 6 of the B‐sheet (B6) in the A1AT structure that are known to play important roles in protein folding, polymerization, and secretion of A1AT (Fraizer, Harrold, Hofker, & Cox, 1989; Graham et al., 1989). The deletion of Phe52 was related to A1AT aggregation in the hepatocytes and consequently reduced the serum A1AT concentration to 18 mg/dl (Matsunaga et al., 1990). The S53F variant (Siiyama variant), resulting in three consecutive Phe residues (Phe51‐Phe52‐Phe53), caused the formation of liver inclusions as a result of polymerization (Lomas, Finch, Seyama, Nukiwa, & Carrell, 1993; Miranda et al., 2010; Seyama et al., 1991). F51C, a mutant obtained from random mutagenesis followed by screening, stabilized A1AT polymerization and slowed down the thermal deactivation rate of A1AT by 10‐fold (Kwon, Kim, Shin, & Yu, 1994; Kwon & Yu, 1997). Another recombinant mutant, F51L, also exhibited similar characteristics of F51C (Dafforn, Mahadeva, Elliott, Sivasothy, & Lomas, 1999; Elliott, Lomas, Carrell, & Abrahams, 1996; Lee, Park, & Yu, 1996; Ryu, Choi, Kwon, Lee, & Yu, 1996). Additionally, F51L increased the secretion level of Z and S_iiyama_ abnormal type of A1AT by 3‐fold and 5‐fold, respectively (Sidhar, Lomas, Carrell, & Foreman, 1995).

Interestingly, there is a SNP of A1AT at Phe51 substituted to hydrophilic amino acid Ser (refSNP number: rs369966794 and mutation nomenclature: NC_000014.9:g.94383014A>G). Notably, the F51S variant gained a new N‐glycosylation site at Asn49 (Asn49‐Ile50‐Ser51). While previous studies of F51C and F51L have focused on the effects of Phe51 in A1AT polymerization, no attention has been paid to the roles of additional N‐glycosylation in F51S mutants. The structure of A1AT suggests that there is a cluster of aromatic‐aromatic interactions among Phe51, Phe190, Phe372, and Phe384. The present study was conducted to investigate the effects of a rare SNP in *SERPINA1* that is independent of known causative variants of A1AT deficiency. We hypothesize that the F51S mutation with an additional glycan moiety may break the hydrophobic interactions between Phe51 and the flanking residues, thereby changing the conformation and stability of the A1AT variant.

## 2 MATERIALS AND METHODS

### 2.1 Selection of SNPs introducing an additional N‐glycosylation

Human A1AT SNP data (2,523 A1AT SNPs in total) were obtained from the SNP database of the National Center for Biotechnology Information (http://www.ncbi.nlm.nih.gov/snp). Intronic mutations or reference alleles were excluded from this study. Among 90 missense variants, the alleles that gain additional glycosylation sites (Asn‐X‐Ser/Thr, where X could be any amino acid except proline (Kornfeld & Kornfeld, 1985)) were selected for further analysis. The previously studied SNPs were also excluded from this study. The glycosylation potentials of the SNPs were predicted using NetNGlyc 1.0 server (http://www.cbs.dtu.dk/services/NetNGlyc).

### 2.2 Site‐directed mutagenesis

Human A1AT cDNA (GenBank accession number: NM_000295) was purchased from the Korea Human Gene Bank (Daejeon, South Korea). The hA1AT gene was subcloned into a TA vector (Enzynomics, South Korea) using two restriction sites of *Hind* III and *Xho* I. Site‐directed mutagenesis was conducted for the F51S mutation using an EzChange site‐directed mutagenesis kit according to the manufacturer's instructions (Enzynomics). The primers used for polymerase chain reaction were: forward primer, 5′‐caccaatatctCcttctccccagtg‐3′ and reverse primer, 5′‐gagaagGagatattggtgctgttggac‐3′. The mutated nucleotides are shown in capital letters. The sequences of constructs were confirmed by DNA sequencing.

### 2.3 Protein expression and purification

The CHO‐K1 cells, which were grown in Dulbecco's Modified Eagle's Medium (Lonza) supplemented with 10% of fetal bovine serum (Gibco) and penicillin (Lonza), were transfected with plasmid DNA using polyethylenimine (Polysciences) as previously described (Xie, Xinyong, Xianjin, & Yayu, 2013). The condition of transfection and expression was optimized to give the highest level of expression as previously described (de los Milagros Bassani Molinas, Beer, Hesse, Wirth, & Wagner, 2014; Xie et al., 2013). The secreted A1AT protein was purified to homogeneity using Alpha‐1 Antitrypsin Select affinity chromatography (GE Healthcare) according to the manufacturer's instructions (Figure S1). Briefly, culture supernatant was diluted with phosphate‐buffered saline (PBS) and loaded onto Alpha‐1 Antitrypsin Select resin equilibrated with PBS buffer. The recombinant protein was eluted by step elution using 1 M MgCl_2_ in 20 mM Tris⋅HCl buffer (pH 7.4). Pooled protein fractions were dialyzed against PBS and concentrated by Amicon centrifugal filters (Figure S1). All purified proteins were filtered using 0.45 µm syringe filters (Millex‐GP) and stored at −80°C.

### 2.4 SDS‐PAGE and Western blotting

To analyze the purity of A1AT protein, approximately 7 µg of purified protein was loaded onto a 10% SDS gel, after which it was stained with Coomassie Brilliant Blue (Thermo Fisher Scientific). To investigate the secreted level of A1AT variant from CHO cells, all proteins in 10 µl of the culture supernatant were run on a 10% SDS gel using SDS‐polyacrylamide gel electrophoresis, then transferred to a PVDF membrane (Roche) for Western blotting. The membrane was incubated in blocking buffer (4% w/v BSA, 0.1% v/v Tween 20 in Tris‐buffered saline) followed by incubation with rabbit anti‐A1AT antibody (Sigma) overnight at 4°C. The membrane was incubated with peroxidase conjugated goat anti‐rabbit secondary antibody (Santa Cruz Biotechnology) in blocking buffer for 1 hr at room temperature, after which immunoreactive bands were visualized using chemiluminescence (Thermo Fisher Scientific) combined with detection by X‐ray film in a dark room.

### 2.5 Enzymatic deglycosylation

To remove N‐glycans, 0.2 µg of purified protein of wild‐type A1AT and F51S variant were boiled in glycoprotein denaturing buffer for 10 min and treated with 50 U of PNGase F (New England Biolabs) in the presence of 1% NP‐40 at 37°C for 1 hr. The cleavage of N‐glycans was confirmed by SDS‐polyacrylamide gel electrophoresis and Western blot analysis.

### 2.6 Trypsin inhibitory assay

Porcine pancreatic trypsin (Sigma) and its substrate N_α_‐Benzoyl–L‐arginine ethyl ester hydrochloride (Sigma) were used to measure the inhibitory activity of A1AT. Briefly, 300 ng of trypsin was incubated with or without 600 ng of A1AT in 40 µl of reaction buffer (15 mM Tris⋅HCl, pH 7.4, 100 mM NaCl, and 0.01% Triton X‐100) at 37°C for 30 min. After incubation, the solution was mixed with 960 µl of 0.25 mM α‐Benzoyl–L‐arginine ethyl ester hydrochloride in reaction buffer at room temperature. The absorbance of the reaction mixture at 405 nm was measured at 0 min and 5 min after reaction and the change in absorbance was used to calculate the remaining trypsin activity.

### 2.7 Protein thermal shift analysis

To determine the melting temperature at which 50% of the protein is unfolded, thermal shift analysis was conducted using a real‐time PCR instrument (Applied Biosystems) with SYPRO orange dye (Life Technologies). Purified protein of the A1AT variant was mixed with SYPRO orange dye in 0.1 M Tris⋅HCl buffer (pH 7.0) to a final concentration of 0.3 µg/µl. The fluorescence signal was obtained from the protein and dye mixture when the temperature increased from 25°C to 99°C. The thermal denaturation curve of each protein was used to calculate T_m_ values utilizing the Protein Thermal Shift Software v1.3 (Applied Biosystems).

### 2.8 Acrylamide‐induced quenching of Trp fluorescence

To measure the conformational flexibility of WT and F51S, acrylamide‐induced quenching of Trp and Tyr fluorescence was measured using a Scinco FS‐2 fluorescence spectrometer. A fixed concentration of 0.1 µg of each protein was prepared in increasing concentrations of acrylamide (0–0.5 M) in Tris⋅HCl buffer (pH 7.0), after which the fluorescence emission spectra of the protein–acrylamide mixture was measured at 25°C and an excitation wavelength of 280 nm. Quenching data were plotted and presented as the ratio of intrinsic fluorescence intensity (F_0_) to the fluorescence intensity with 0–0.5 M acrylamide (F).

## 3 RESULTS

### 3.1 Expression of F51S in CHO‐K1 cells

There were two potential N‐glycosylation SNPs among hA1AT variants: F51S (refSNP number: rs369966794) and P369S (refSNP number: rs61761869). Because P369S SNP was reported to be related to A1AT deficiency (Fraizer et al., 1989), we used F51S SNP for this study. The glycosylation potential of F51S mutant determined via the NetNGlyc server was 0.46 (the maximum potential was 1 and the threshold for glycosylation was 0.5).

**Figure 1:**
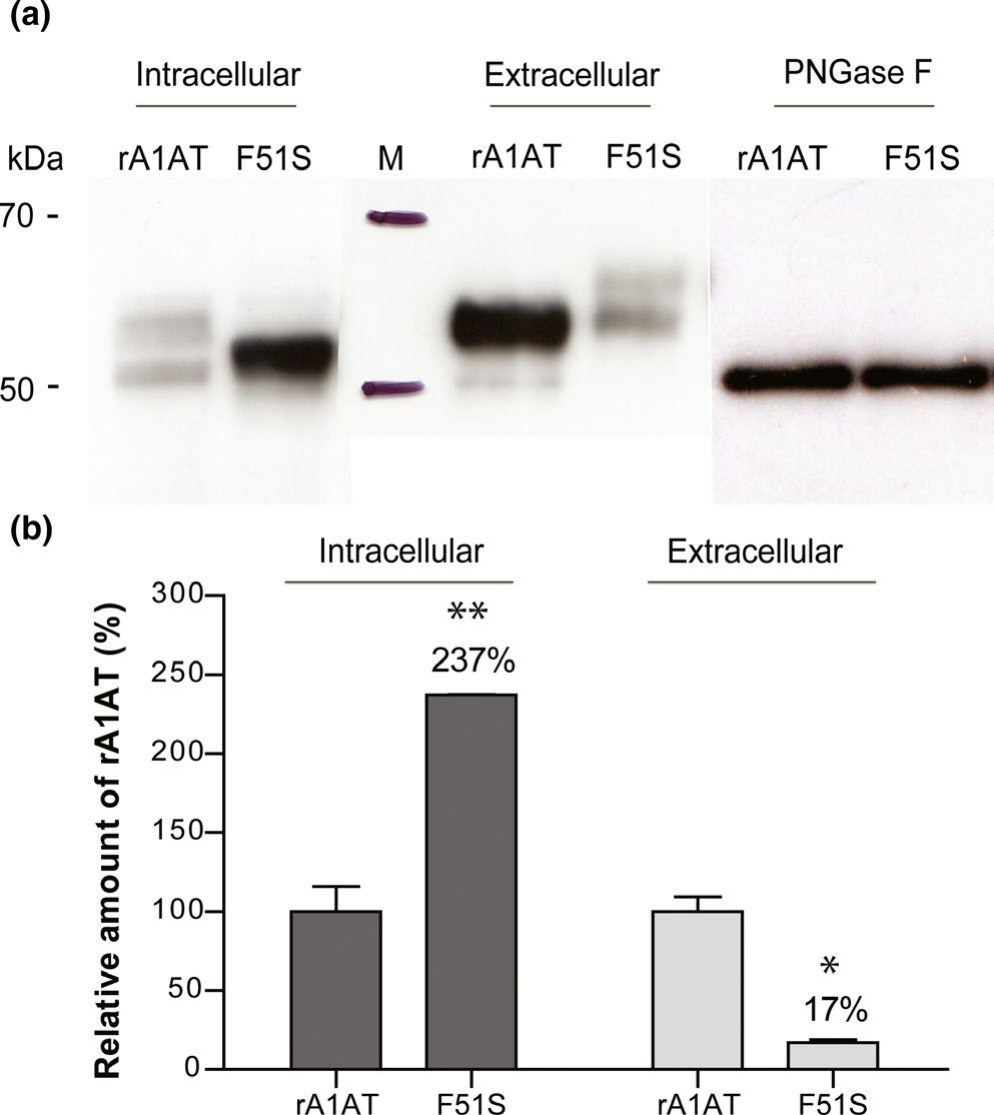
Expression of WT and F51S. (a) The Western blot data show the level and molecular weight of WT and F51S inside the transfected CHO‐K1 cells and secreted in media. The molecular weight of deglycosylated WT and F51S are similar upon PNGase F treatment, approximately 50 kDa. (b) The level of A1AT proteins was calculated as a bar graph from Western blot images using the ImageJ software. Data correspond to the mean ± *SD*. Two‐tailed unpaired *t* test between F51S and rA1AT were conducted (*p < 0.05, **p < 0.01)

The F51S variant was expressed in CHO‐K1 cells as a soluble protein, but was accumulated inside cells. Figure 1a shows two states of intracellular WT, un‐glycosylated WT (upper band) and glycosylated WT (lower band), which were present in similar proportions (52% and 48% respectively). Moreover, most of the intracellular F51S protein (98%) was un‐glycosylated. The data suggested that only a small amount of intracellular F51S (2%) had undergone post translational modification processes and been secreted. Consistent with the cell extract data, the secreted A1AT of F51S in culture supernatant was 6‐fold lower than that of WT (Figure 1b). The extracellular F51S protein appeared on an SDS‐gel with a higher molecular weight than the WT because of additional N‐glycosylation (Figure 1a and Figure S2). However, this difference disappeared when the proteins were treated with PNGase F (Figure 1a).

### 3.2 The inhibitory activity of F51S variant

**Figure 2:**
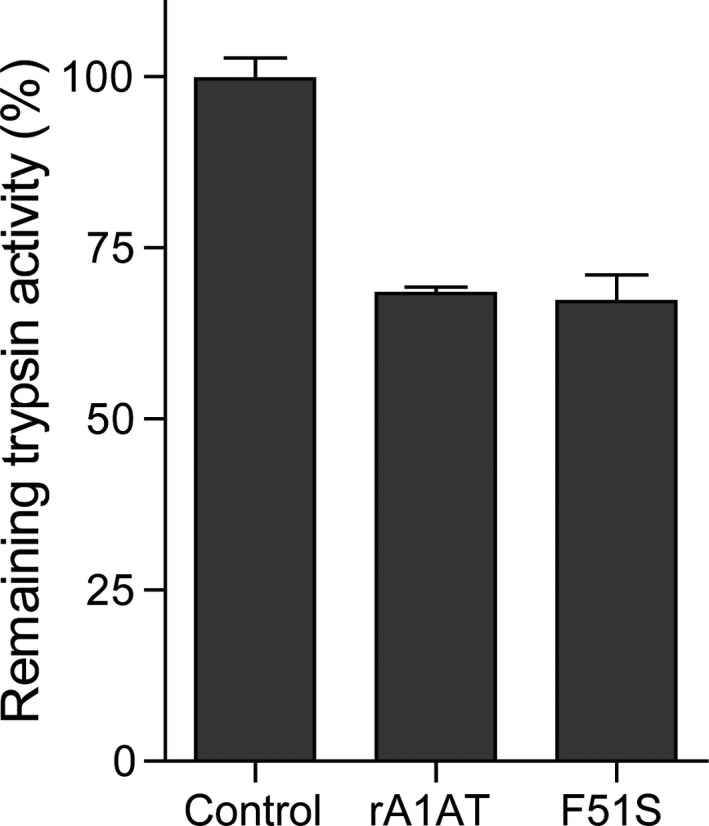
Inhibitory activity of F51S compared with WT. The trypsin inhibitory activities are shown upon incubation of WT and F51S (1:1 molar ratio with trypsin) at 37°C for 30 min. The activity of trypsin without inhibitor was 100% (control). Experiments were repeated three times. Data correspond to the mean ± *SD*. One‐way ANOVA and Tukey's multiple comparison test for F51S and rA1AT; no significant difference was found (*p* > 0.05, *q* = 0.4894)

To investigate whether an additional N‐glycosylation at Asn49 affected the inhibitory activity of A1AT, the trypsin inhibitory activity was measured at a 1:1 molar ratio of A1AT and trypsin. The activity of F51S variant was similar to that of WT (Figure 2). The results suggested that the additional N‐glycosylation at Asn49 did not affect the antitrypsin activity of F51S.

### 3.3 Prevention of polymerization by F51S mutation

**Figure 3:**
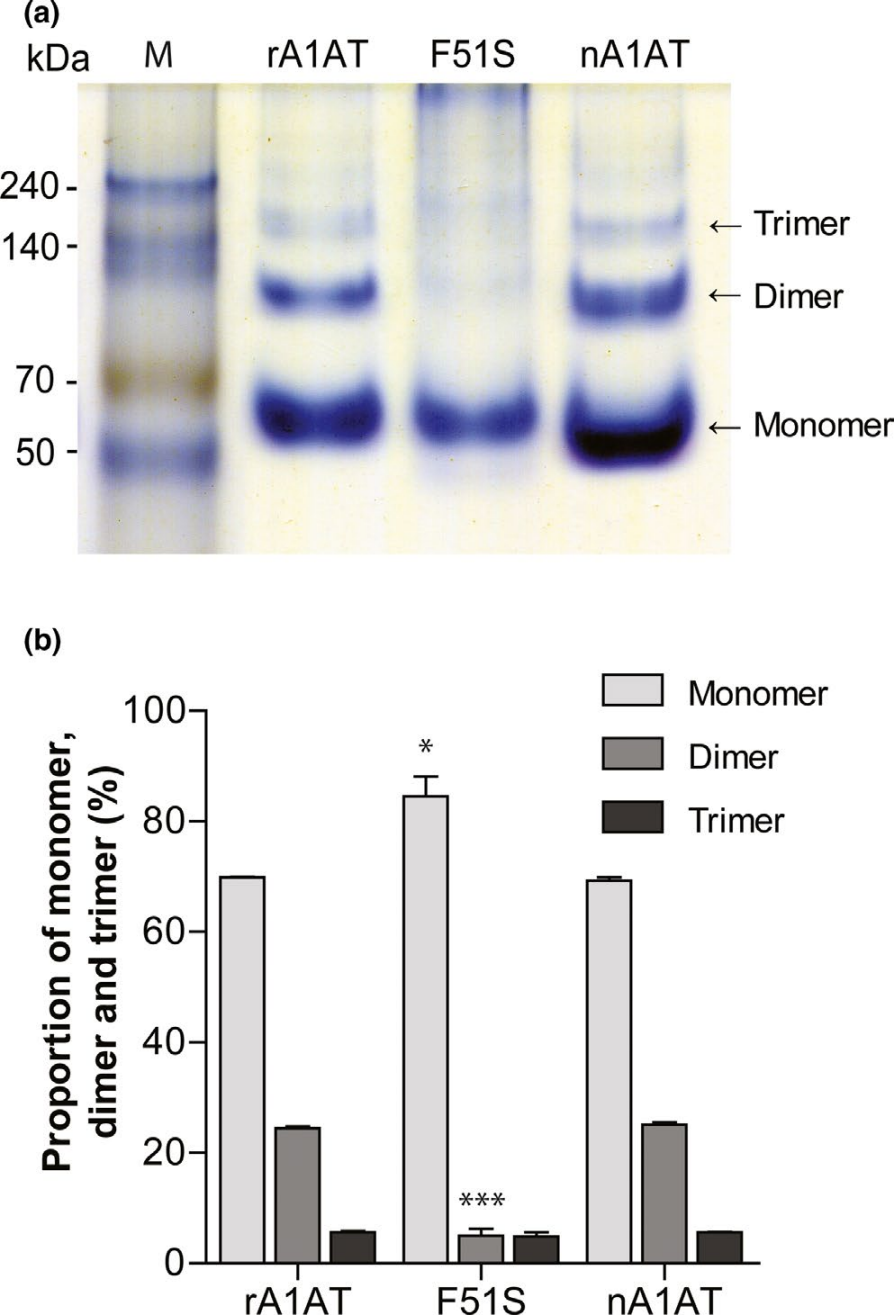
The polymerization level of A1AT variants. (a) The native 10% acrylamide gel separated monomers, dimers, and trimers of rA1AT, F51S, and nA1AT. (b) The proportion of monomers and polymers of the above three A1AT variants was built based on native gel images using the Image J software. Experiments were repeated three times. Data correspond to the means ± *SD*. One‐way ANOVA test showed a group effect (*F* = 209.5; *p* < 0.001) and Tukey's multiple comparison test between each type of F51S (monomer, dimer, or trimer) and the corresponding rA1AT indicated that the difference was significant (* *p* < 0.05, *** *p* < 0.001)

The polymer proportions of F51S and the native A1AT were determined as shown in Figure 3. Similar to the F51L and F51C mutants, the F51S mutant showed a decrease in the ratio of dimers and trimers compared with native plasma A1AT (nA1AT) and recombinant A1AT (rA1AT). Approximately 90% of F51S existed in a monomer state. When compared with F51S, the proportions of rA1AT and nA1AT polymers were three times higher.

### 3.4 Thermal stability and conformational flexibility

**Figure 4:**
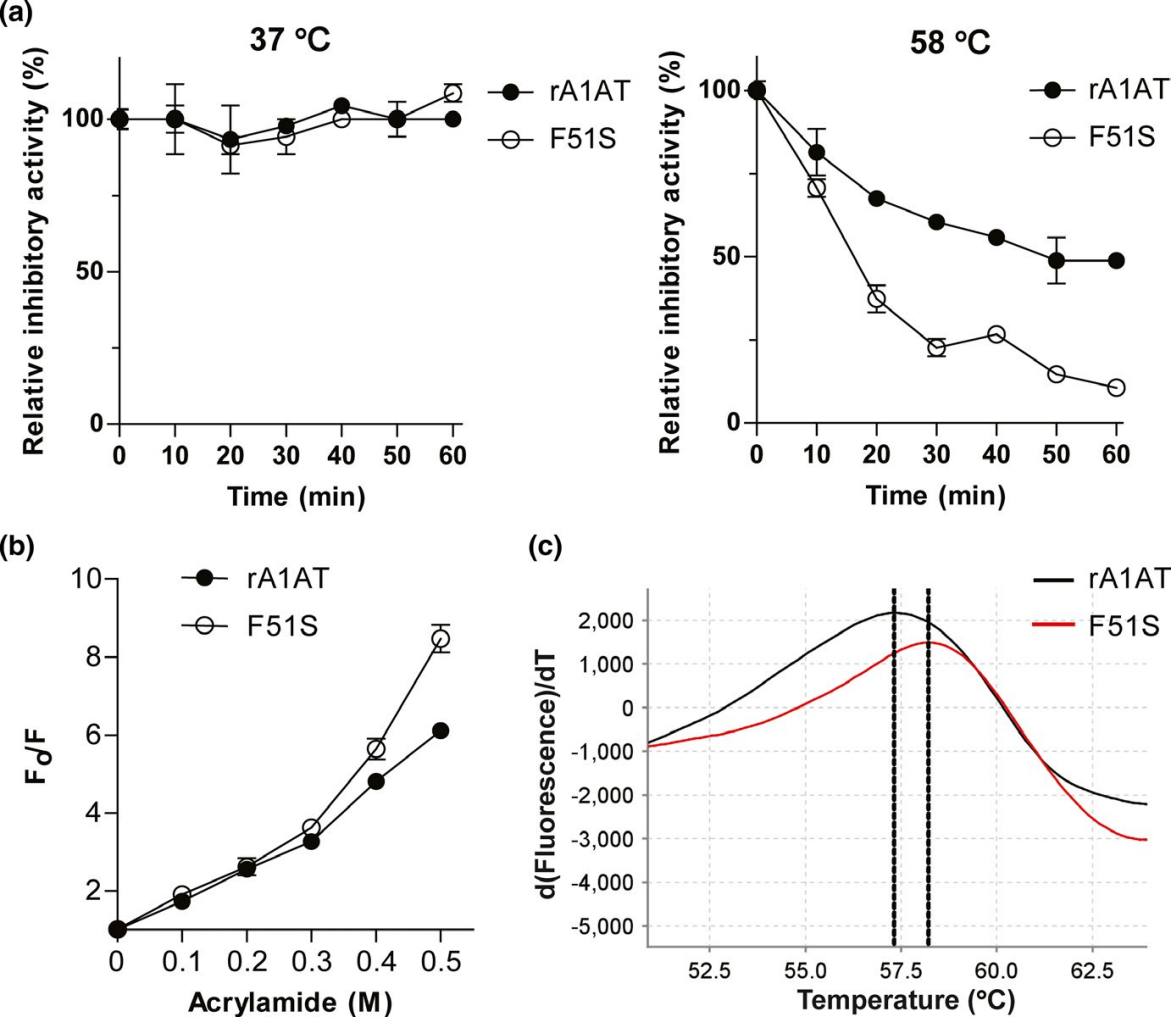
The thermal stability of A1AT at 37°C and 58°C (a), acrylamide‐induced fluorescence quenching (b) and the thermal denaturation curves (Protein Thermal Shift Software v1.3) (c) of WT and F51S proteins. The thermal stability of F51S and WT at 37°C were identical, whereas there was a dramatic loss in thermal stability of F51S at 58°C compared with WT

To investigate whether an additional N‐glycosylation affected the thermal stability of the F51S variant, the remaining trypsin inhibitory activity was measured after incubation at 37°C or highly polymerized temperature (58°C) for the indicated time. Interestingly, the thermal stability of F51S and WT at 37°C were similar. However, there was a sharp decrease in the thermal stability of F51S at 58°C when compared with that of WT (Figure 4a). The time to loss of half of inhibitory activity at 58°C of WT and F51S was 60 min and 20 min, respectively. This loss of thermal stability could be a consequence of a more flexible conformation of F51S.

To determine the relationship between thermal stability and structure, protein thermal shift analysis and acrylamide‐induced quenching were conducted. The melting temperature of F51S mutant was 58.2°C, a slight increase from the T_m_ value of the WT (57.3°C) (Figure 4c), suggesting that the F51S mutants have increased overall thermal stability. However, this change in T_m_ value was not as high as the data observed for the F51L mutant. The T_m_ of the F51L increased 4°C (from 59.6°C to 63.6°C) compared with the WT (Dafforn et al., 1999). Lee et al. also reported that F51L increased the change in free energy of stabilization (ΔΔG) to 2.1‐fold higher than WT (Lee et al., 1996). The flexibility of F51S was evaluated by acrylamide quenching of Trp and Tyr fluorescence upon excitation at 280 nm. The Stern‐Volmer plot indicated that F51S is more flexible than WT (Figure 4b). Three of the four inner Tyr residues of A1AT are located near the Phe51 residue; hence, the additional glycosylation in the F51S mutant may disrupt hydrophobic interaction and increase the local flexibility of the variant. These results showed that the substitution of Ser for Phe51 slightly strengthened the conformation toward preventing polymerization of A1AT; however, it reduced the thermal stability of the protein.

## 4 DISCUSSION

The SNPs with minor allele frequencies (MAFs) of less than 0.1 are normally excluded from rare allele studies even though most of the human SNPs are rare (MAFs < 0.05) as the studies require a large sample size, up to millions of people. However, recent studies have indicated that rare alleles play an important role in the risk of common diseases and tend to have a stronger effect than common SNPs (Gorlov, Gorlova, Frazier, Spitz, & Amos, 2011; Gorlov, Gorlova, Sunyaev, Spitz, & Amos, 2008). Therefore, an alternative approach that reduces effort and budget but still provides initial satisfactory conclusions of the effects of the rare alleles is highly necessary. We investigated the abnormal characteristics of a minor allele of SNP rs369966794 using biochemical experiments. Subsequently, our data suggest that this rare allele is related to A1AT deficiency in humans.

The polymerization of A1AT is related to the reduced level of A1AT. The Z mutation (E342K), which increased the polymerization 19‐fold when compared with WT (Dafforn et al., 1999), led to emphysema and liver disease (Lomas, Evans, Finch, & Carrell, 1992). The H334A mutant, which rapidly formed polymers that accumulated within the endoplasmic reticulum, was found in a prolonged jaundice infant (Miranda et al., 2010). However, the polymerization could reduce the diffusion ability in plasma and the shelf‐life of commercial A1AT (Dafforn et al., 1999; Lomas, Elliott, Chang, Wardell, & Carrell, 1995)). The relationship between polymerization and A1AT inhibitory activity is still unknown and the mechanism of A1AT polymerization remains controversial (Dafforn et al., 1999; Ekeowa et al., 2010; Gooptu et al., 2000; Lomas et al., 1992). The A1AT polymerization has a similar mechanism of Alzheimer's disease. Several studies investigating methods of inhibiting A1AT polymerization have been reported (Alam, Wang, Janciauskiene, & Mahadeva, 2012; Bottomley & Tew, 2000; Burley & Petsko, 1988; Parfrey et al., 2003). Therefore, the prevention of A1AT polymerization by F51S SNP could be an advantage for patients (Figure 3). There is convincing evidence regarding the role of Phe51 in A1AT polymerization. The F51L mutant lowered the level of energy in the native state that is kinetically trapped in the folding reaction (Lee et al., 1996) and retarded the conversion of the native inhibitors into the stable latent conformation. A complex of acetate ion and A1AT was made to prevent A1AT polymerization (Pearce et al., 2008). The acetate ion binds to the B‐sheet of A1AT, in which Phe51 is located.

**Figure 5:**
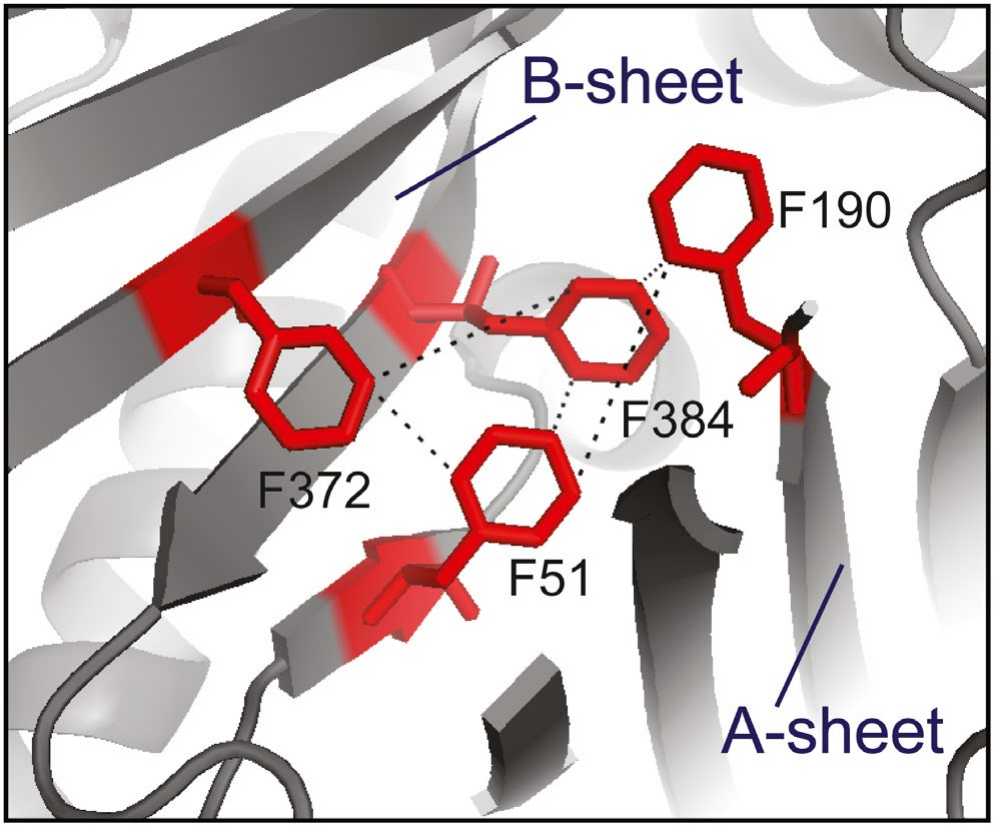
The aromatic–aromatic interactions surrounding Phe51 of A1AT. A crucial Phe cluster forms a hydrophobic core in the A1AT. The pi–pi interactions between Phe residues surrounding Phe51 were built by YASARA View (Krieger & Vriend, 2014) with a range of distance of 3.4–6.5 Å (Burley & Petsko, 1988). The figure was generated from the crystal structure of intact native A1AT (PDB ID: 3NE4) using PyMOL (Version 1.3)

The important role of aromatic‐aromatic interactions in maintaining protein structure has been investigated by several research groups (Aravinda et al., 2003; Lanzarotti, Biekofsky, Estrin, Marti, & Turjanski, 2011; Madhusudan Makwana & Mahalakshmi, 2015). On average, 60% of aromatic side chains in proteins are involved in aromatic pairs, 80% of which form networks of three or more interacting aromatic side chains (Burley & Petsko, 1988). An investigation of Phe clusters inside the villin headpiece subdomain revealed that it is critical to attain the native fold and maintain thermal stability of the protein (Madhusudan Makwana & Mahalakshmi, 2015). When Phe was individually substituted with Leu, the substitutions resulted in destabilization of villin, wherein the mid‐point of thermal denaturation drops from 70°C to 35–50°C. The structure of the conserved Phe cluster forms of the villin headpiece subdomain are similar to the structure of the Phe cluster surrounding Phe51 in the A1AT core (Figure 5). The Phe51 residue is also located in a conserved Phe cluster (Phe370, Phe372, Phe381, and Phe51) that form aromatic–aromatic interactions. Therefore, substitution of Phe51 to another hydrophilic or non‐aromatic residue could break the hydrophobic interactions. Notably, there is an interaction between Phe51 in the B‐sheet and Phe372 in the A‐sheet. The substitution of Phe to Ser and insertion of an additional glycan may not only break some inter‐strand interactions in parallel β‐sheets, but can also reduce the interaction between B‐ and A‐sheets. The highly flexible nature of the F51S (Figure 4b) is consistent with structure model analysis. The change in conformation of F51S may lead to a significant reduction in the secretion level (Figure 1); however, the activity of A1AT was maintained (Figure 2). Changes in conformation were related to the reduced ability to bind to the transport receptor ERGIC‐53 and therefore significantly lowered the secretion level of A1AT (Nyfeler et al., 2008).

The results show that F51S had a low secretion level (Figure 1) and decreased thermal stability at 58°C (Figure 4a), while similar Phe51 substitutions (F51C and F51L) increased the secretion level and thermal stability (Dafforn et al., 1999; Kwon & Yu, 1997). Differences in F51S from other Phe51 variants include that F51S gained a new N‐glycosylation site and affected the hydrophobic characteristic of the region. Florescence quenching data suggested that F51S is more flexible than WT (Figure 4b). The change in conformation of F51S prevented A1AT polymerization, but also reduced its thermal stability.

This study proposed an approach that may facilitate the detection of risk‐associated rare SNPs. The effects of a genotype on phenotype not only depend on genotype itself, but also depend on the interactions between a number of different genes and the environment. Therefore, the results of this study need to be validated clinically. The GenBank database can be used to focus on patients with rare SNPs to analyze their phenotype and investigate the relevance of rare SNPs to the risk of target disease.

In conclusion, the study presented herein provides evidence that the F51S mutation significantly decreased the secretion of A1AT in CHO‐K1 cells and the thermal stability of A1AT, suggesting that F51S variant could lead to A1AT deficiency in humans.

## CONFLICT OF INTEREST

The authors declare that they have no conflicts of interest.

## AUTHOR CONTRIBUTIONS

HNT, SHL, and CL designed the project. HNT, SHJ, and CL analyzed the data. HNT and CL wrote the manuscript. All authors read and approved the final manuscript.
