# Supplementary material for: Functional characterization of a SNP (F51S) found in human alpha 1‐antitrypsin
Source: Mol Genet Genomic Med. 2019 Jun 28;7(8):e819. doi: 10.1002/mgg3.819 (PMC6687665; doi:10.1002/mgg3.819)

**Supporting Information For:**

**Functional characterization of a SNP (F51S) found in human alpha 1-antitrypsin**

**Hong-Nhung Trinh, Sei-Heon Jang, and ChangWoo Lee***

Department of Biomedical Science and Center for Bio-Nanomaterials, Daegu University,

Gyeongsan 38453, South Korea

***Correspondence:** ChangWoo Lee, Department of Biomedical Science, Daegu University, Gyeongsan 38453, South Korea. Tel: +82-53-850-6464; Fax: +82-53-850-6469; E-mail address: [leec@daegu.ac.kr](mailto:leec@daegu.ac.kr)

**Figure S1**


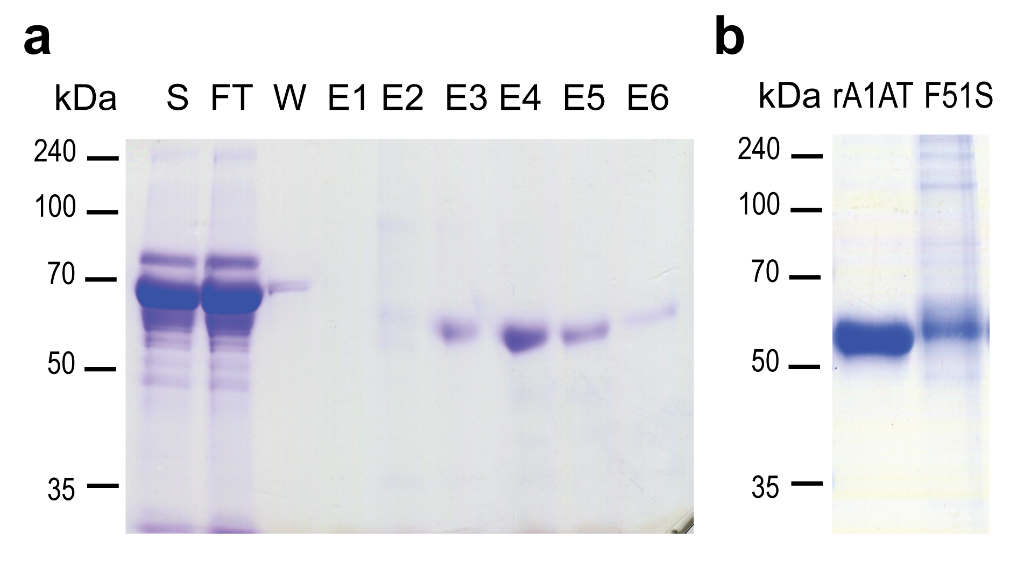


**Figure S2**


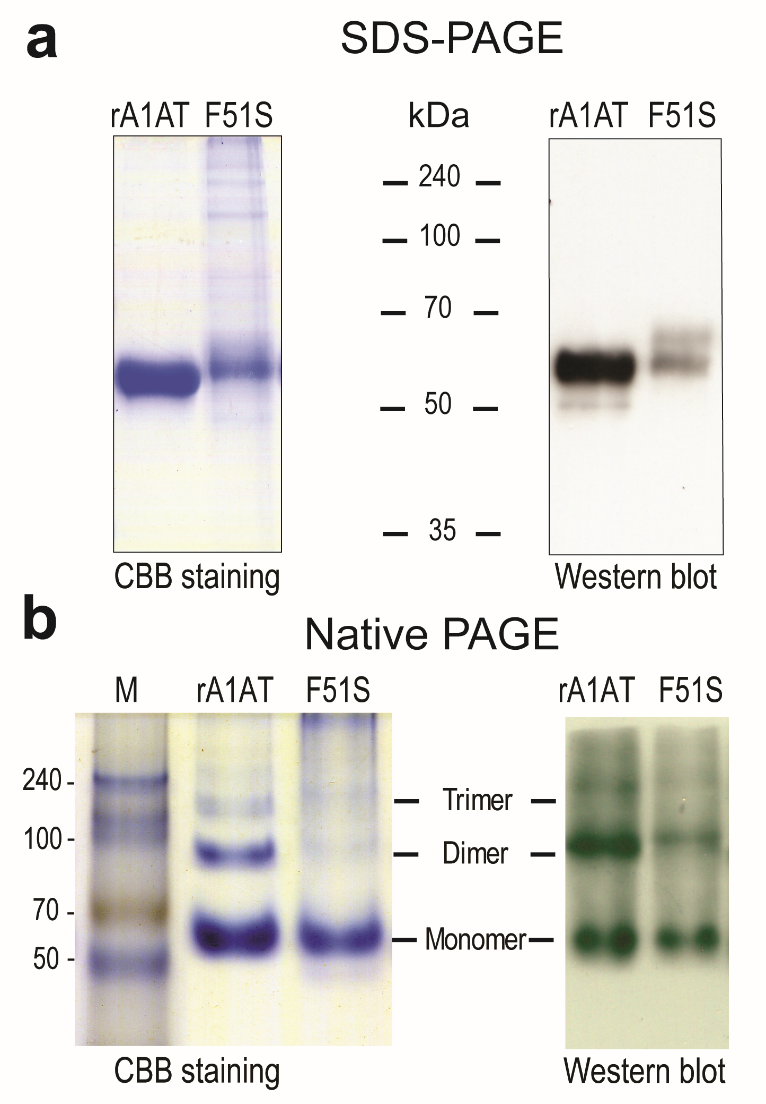

Supplement: Supplementary file 1 [file MGG3-7-e819-s001.docx]
